# P-2312. Impact of a Short Course Post-Operative Antibiotic Prophylaxis Duration on Nephrotoxicity in Lung Transplant Recipients

**DOI:** 10.1093/ofid/ofae631.2464

**Published:** 2025-01-29

**Authors:** Paige Stratton, Rachel M Kenney, Michael Veve, Mary Grace Fitzmaurice, George J Alangaden, Domingo Franco-Palacios

**Affiliations:** Henry Ford Hospital, Royal Oak, Michigan; Henry Ford Hospital, Royal Oak, Michigan; Henry Ford Health, Detroit, Michigan; Henry Ford Hospital, Royal Oak, Michigan; Henry Ford Health, Detroit, Michigan; Henry Ford Hospital, Royal Oak, Michigan

## Abstract

**Background:**

Vancomycin plus an antipseudomonal β-lactam is used as lung transplant surgical prophylaxis, but an optimal post-operative duration is not defined. The study objective was to assess the impact of a shortened antibacterial surgical infection prophylaxis (SIP) duration on post-operative nephrotoxicity in lung transplant recipients.

Table 1

**Figure:**
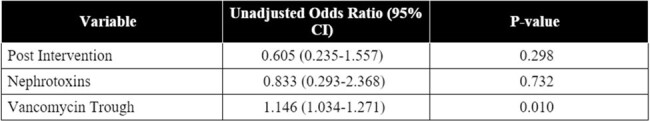

Variables associated with AKI

**Methods:**

IRB approved quasi experiment of lung transplant recipients who received SIP from 1/1/2016-9/30/2020 (pre-group) to 10/1/2020-7/31/24 (post-group). Intervention: implementation of shortened SIP duration to 72-hours of cefepime and vancomycin post-operatively. Inclusion: eGFR >30 mL/min/1.73m2 between transplant day ±2. Exclusion: renal replacement ≤ 3-months, simultaneous organ transplants, donor bronchi with Gram-positive bacterial growth, or new COVID-19 infection between transplant day ±7. The primary endpoint was the incidence of acute kidney injury (AKI), defined by the KDIGO criteria, while receiving post-operative vancomycin up to 14 days. Secondary endpoints were vancomycin consensus guideline (VCG) AKI definition at 14-days and time to AKI.

**Results:**

77 patients were included: 45 (58%) pre-, 32 (42%) post-intervention. 73% vs. 47% male (p=0.018). 49% vs. 34% (p=0.205) KDIGO AKI while on vancomycin. Post-group associated with approximately 40% decreased odds of developing KDIGO AKI (Table 1). Secondary endpoints: 33% vs. 19% VCG AKI (p=0.157). Median time to AKI was three days, with no differences detected between groups and AKI definitions. Similar rates of C. diff infection, positive bacterial respiratory cultures, new multidrug resistant organisms, surgical site infections. 57.8% vs. 84.4% patients received treatment for new pneumonias after SIP completion (p=0.013), with one new MRSA pneumonia in the pre-group and one in the post-group.

**Conclusion:**

Implementation of a shortened antibacterial prophylaxis protocol for lung transplant resulted in numerically fewer AKIs. Maturation, regression to the mean, and reliance on manual chart review are all limitations of this retrospective study. Ongoing analysis of this intervention, such as with multicenter prospective studies, can help to characterize the decreased nephrotoxicity risk with shorter course prophylaxis post-transplant.

**Disclosures:**

Rachel M. Kenney, PharmD, BCIDP, Medtronic Inc: Spouse is an employee, stockholder

